# Phenotypic Detection of Antimicrobial Resistance Patterns in Microorganisms Isolated From the Primary Molars of Pediatric Dental Patients Undergoing Pulp Therapy

**DOI:** 10.7759/cureus.106128

**Published:** 2026-03-30

**Authors:** Deepak Khandelwal, Rishi Tyagi, Amit Khatri, Kirti Nirmal, Padma Yangdol, Swati Nirmal, Aman Kumar, Misbah Shaikh, Nimisha Jain

**Affiliations:** 1 Dentistry, University College of Medical Sciences, Delhi, IND; 2 Microbiology, University College of Medical Sciences, Delhi, IND

**Keywords:** antibiotic therapy, antimicrobial resistance, dental, dental therapy, endodontic treatment, microorganisms, pediatric dental patients, primary molar, pulp therapy

## Abstract

Background

Pulp necrosis is a prevalent complication following dental caries in primary dentition. Certain bacteria exhibit increased adaptability to adverse conditions and possess virulence factors that enable more effective colonization of dental tissues. The invasion of the root canal system (RCS) by pathogenic microorganisms, compounded by the rising concern of antimicrobial resistance (AMR), emphasizes the necessity for a culture-driven and evidence-based approach to antibiotic selection in pediatric endodontics, wherein the therapeutic efficacy of root canal treatment is inherently dependent on the comprehensive elimination of endodontic microbiota.

Aim

To isolate, identify, and analyze the antimicrobial susceptibility and resistance profiles of microorganisms associated with endodontic infections in the deciduous teeth of children undergoing pulp therapy.

Materials and methods

One hundred systemically healthy children aged four to nine years with carious teeth and without direct endodontic exposure were enrolled following Strengthening the Reporting of Observational studies in Epidemiology (STROBE) guidelines at the Department of Pediatric and Preventive Dentistry, University College of Medical Sciences (UCMS) & Guru Teg Bahadur Hospital (GTBH), Delhi, India. Root canal samples from infected or necrotic primary teeth were aseptically collected and cultured on blood, chocolate, and MacConkey agar. Bacterial isolates were identified via Gram staining and standard biochemical assays. Statistical evaluation was conducted using IBM SPSS Statistics for Windows, Version 21 (Released 2012; IBM Corp., Armonk, New York, United States).

Results

Bacterial growth was detected in 68 samples (68%), predominantly involving Gram-positive cocci (n=48; 48%). Enterococcus faecalis (n=25; 25%) was the most common isolate, followed by Staphylococcus aureus (n=17; 17%) and a smaller proportion of Gram-negative bacilli, including Enterobacter cloacae, Escherichia coli, and Klebsiella pneumoniae. All isolates showed complete sensitivity to vancomycin, teicoplanin, linezolid, amikacin, and imipenem, while variable resistance to macrolides, clindamycin, fluoroquinolones, and β-lactams was observed. No extended-spectrum β-lactamase (ESBL) or carbapenem-resistant strains were identified.

Conclusion

Enterococcus faecalis remains the predominant pathogen in pediatric endodontic infections, with its increasing multidrug resistance presenting significant therapeutic challenges. Nevertheless, β-lactam and glycopeptide antibiotics continue to demonstrate reliable clinical efficacy. These findings highlight the importance of culture-guided therapy and rational antimicrobial stewardship in pediatric dental practice.

## Introduction

Primary teeth are highly susceptible to damage from physical, chemical, and biological sources. Extensive dental caries and traumatic injuries can lead to pulp necrosis, resulting in premature loss of these teeth [1]. Pulp necrosis is a prevalent complication following dental caries in primary dentition, when carious lesions expose the pulp, bacteria from the carious biofilm colonize the dentinal surfaces, leading to the formation of biofilms within the root canal system (RCS) [2]. While any microorganism that enters the RCS has the potential to cause an endodontic infection, certain bacteria exhibit enhanced flexibility to unfavorable conditions and possess virulence factors that enable them to more effectively colonize dental tissues, leading to frequent infections [3,4].

Microbial invasion of the root canal system initiates inflammatory responses driven by bacterial biofilms and virulence factors such as lipopolysaccharides (LPS) and lipoteichoic acid (LTA), leading to pulpal breakdown and periradicular tissue damage [5]. In healthcare, including dentistry, the judicious use of antibiotics is crucial due to the associated risk of antimicrobial resistance. The focused oral sepsis theory suggests that antibiotics are rarely given in dental treatments to mitigate the risk of bacteremia from oral infections or surgical procedures. However, it is essential to avoid giving antibiotic empirically to all patients without first identifying and determining in vitro susceptibility of the implicated microflora. This approach underscores the importance of rational antibiotic use in dentistry to minimize the proliferation of multidrug-resistant bacteria [6,7].

The efficacy of root canal therapy is intrinsically linked to the successful reduction or eradication of the endodontic microbiota. The prescription of antimicrobials should be meticulously based on the bacterial resistance profile, patient-specific factors, and the clinical treatment. An in-depth understanding of the infection's characteristics allows for the precise selection of antibiotics with the appropriate spectrum of dosage and frequency. This methodical approach is crucial for mitigating the emergence and dissemination of antimicrobial resistance, which poses a significant challenge in contemporary clinical practice. Despite extensive research on permanent teeth microbiota, there is limited investigation into primary dentition infections and the effects of dental caries [8,9]. Therefore, the present study was done to identify and characterize the antimicrobial susceptibility and resistance profiles of oral microorganisms isolated from primary endodontic infections in deciduous teeth of pediatric patients undergoing pulp therapy.

## Materials and methods

Selection of patients and clinical cases

A total of 100 systemically healthy children aged four to nine years were recruited from the Unit of Pediatric and Preventive Dentistry, University College of Medical Sciences (UCMS) & Guru Teg Bahadur Hospital (GTBH), Delhi, India, in accordance with the Strengthening the Reporting of Observational studies in Epidemiology (STROBE) guidelines [10]. Ethical approval was obtained from the Institutional Research Ethics Committee (GTBHEC 2024/P-224).

Children presenting with carious teeth without direct endodontic exposure, radiographically intact roots, and clinical signs of irreversible pulpitis or pulp necrosis with or without abscess, requiring endodontic intervention were included in the study. Participants were allocated using a simple randomization method, ensuring unbiased distribution and minimal selection bias. Informed written consent was obtained from parents or guardians prior to participation.

Exclusion criteria comprised teeth close to natural exfoliation and children who had received systemic antibiotics within the preceding three months for any reason.

Specimen sample collection

**Figure 1:**
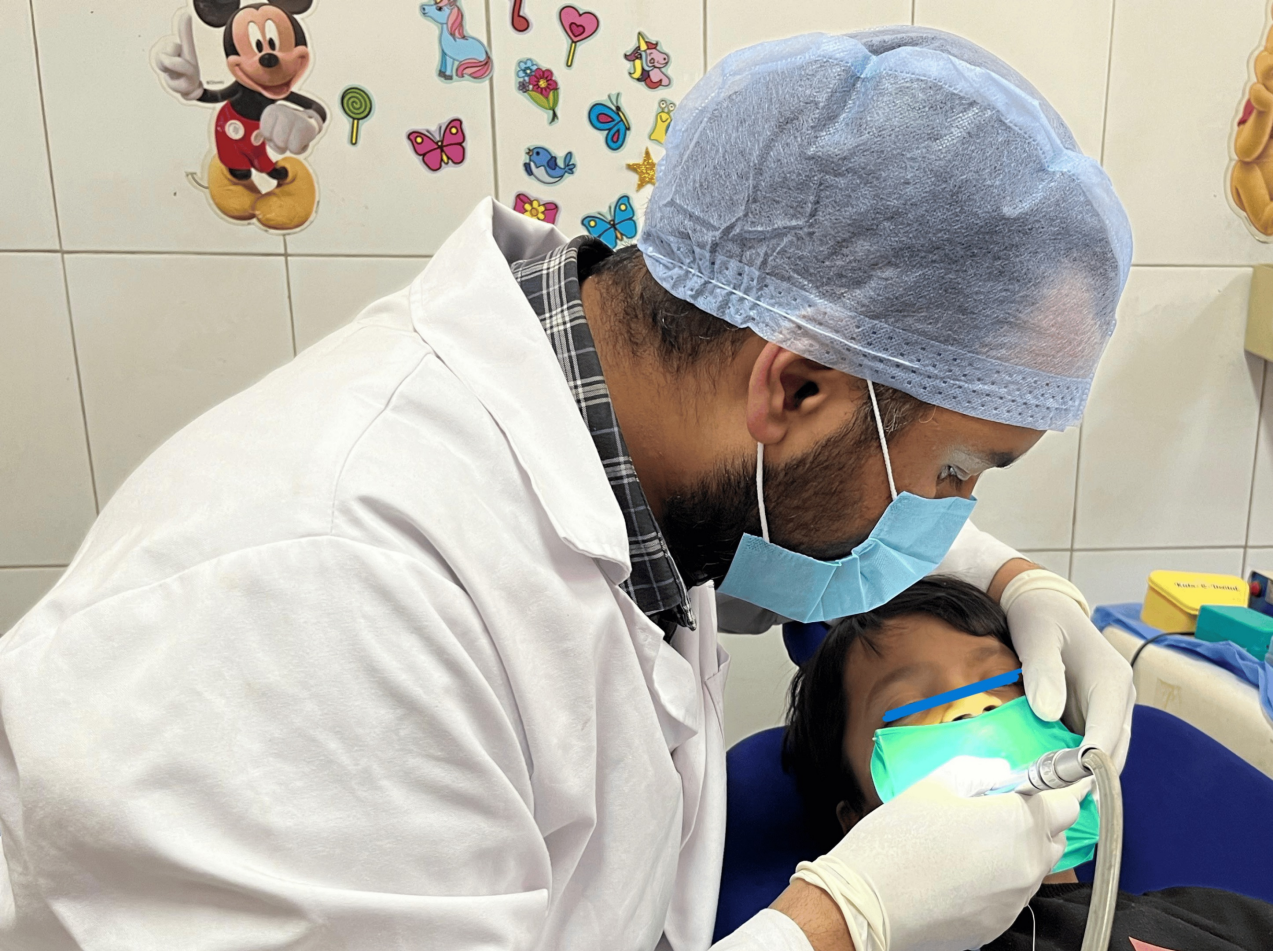
Endodontic procedure performed by the operator

The standard protocol for bacterial sample collection was followed [10]. All endodontic procedures and sampling were performed by a single experienced operator (Figure 1).

The operative field was disinfected using a pre-procedural mouth rinse with 2% chlorhexidine gluconate. Participants were instructed to rinse for 30-60 seconds prior to the procedure to reduce the microbial load. After this, local anesthesia was administered and a rubber dam was secured on the carious teeth. Access cavity preparation was then carried out.

Baseline tissue debris samples were obtained using sterile No. 15 endodontic K-files (Mani Inc., Tochigi, Japan) with discrete up-and-down filing motions. Subsequently, bacterial samples were collected by inserting four absorbent paper points (ISO size #20 and #25; 0.02 and 0.04 taper, Meta Biomed Co. Ltd., Korea) for one minute to the predetermined working length. The paper points were immediately transferred into test tubes containing 1.7-2.5 mL of reduced transport medium (brain-heart infusion broth (BHIB)) and promptly transported to the microbiology laboratory for culture and isolation. Following sample collection, the teeth were treated endodontically and restored with stainless steel crowns.

All clinical procedures and sample collection were completed during a single visit for each participant. The study was conducted over a period of six months, during which samples were collected during routine endodontic procedures with an average of two to three procedures carried out per day.

Microbiology laboratory procedure

Each clinical sample was processed to isolate the causative pathogen, followed by its identification and antimicrobial susceptibility testing (AST). A direct Gram stain was performed from the BHIB. For primary culture, inoculated BHIB was incubated at 37 °C overnight. After incubation, the broth was vortexed, and 1 mL aliquots were inoculated onto chocolate, blood, and MacConkey agar plates. The blood and MacConkey agar plates were incubated aerobically at 37 °C overnight, while the chocolate agar plates were incubated at the same temperature in a candle jar to provide a capnophilic environment. Bacterial isolates were identified based on Gram stain, colony morphology, and standard biochemical tests as per routine laboratory procedures.

Antimicrobial susceptibility testing

The isolated pathogens were subjected to AST using the Kirby-Bauer disk diffusion method on 90 mm Mueller-Hinton agar (MHA) plates. Lawn cultures were prepared, and the following antimicrobial disks were applied. For Gram-positive isolates: Cefoxitin (30 µg), Ciprofloxacin (5 µg), Erythromycin (15 µg), Clindamycin (2 µg), Gentamicin (10 µg and 120 µg for high-level resistance), Tetracycline (30 µg), Cotrimoxazole (1.25/23.75 µg), Chloramphenicol (30 µg), and Linezolid (30 µg). For Gram-negative isolates: Ampicillin (10 µg), Amikacin (30 µg), Tobramycin (10 µg), Amoxicillin-Clavulanate (20/10 µg), Gentamicin (10 µg), Piperacillin-Tazobactam (100/10 µg), Cefotaxime (30 µg), Ceftazidime (30 µg), Ceftriaxone (30 µg), Tetracycline (30 µg), Ciprofloxacin (5 µg), Cotrimoxazole (1.25/23.75 µg), Chloramphenicol (30 µg), Aztreonam (30 µg), Imipenem (10 µg), and Meropenem (10 µg). All MHA plates were incubated aerobically at 37 °C overnight. Inhibition zone diameters were measured and interpreted as susceptible, intermediate, or resistant according to the latest Clinical and Laboratory Standards Institute (CLSI) M100 (34th edition) guidelines [11].

Phenotypic detection of resistance mechanisms

Additional phenotypic tests were carried out to detect specific resistance mechanisms. Among Gram-positive isolates, Staphylococcus aureus was screened for methicillin resistance (MRSA) using the Cefoxitin (30 µg) disk method, while inducible clindamycin resistance (ICR) was identified using the D-test with Erythromycin (15 µg) and Clindamycin (2 µg) disks. Among Gram-negative isolates, extended-spectrum β-lactamase (ESBL) production was screened using Cefotaxime (30 µg) or Ceftazidime (30 µg) disks, tested with and without Clavulanate (10 µg). Detection of carbapenem-resistant Enterobacterales (CRE) was performed using the Carba NP test (RAPIDEC® CARBA NP; bioMérieux, France).

Statistical analysis

Data were obtained and entered into Microsoft Excel (version 13; Microsoft Corp., Redmond, WA, USA) and subsequently analyzed using IBM SPSS Statistics for Windows, Version 21 (Released 2012; IBM Corp., Armonk, New York, United States). For categorical variables, frequencies and percentages were calculated, while continuous variables were summarized using means and standard deviations. Associations between variables were assessed using Kendall’s Tau correlation. Binary logistic regression was employed to evaluate the effects of organism presence, antimicrobial susceptibility, and resistance. All statistical tests were conducted at a 95% confidence interval, and a p-value of <0.05 was considered to be statistically significant.

## Results

The study comprised 100 children aged 4-9 years (mean 6.90 ± 1.47), with 53% females and 47% males. Pain was reported in the vast majority of children (98%). Extraoral swelling was observed in 29% of cases, while intraoral swelling was present in 82% (n=82). Abscess formation was identified in 89% of the sample, and dental caries was present in all children (100%). Tenderness on percussion was noted in 73% of cases. Radiographic evaluation revealed periapical or furcal involvement in 78% and root resorption in 73% of the children (Table 1). 

**Table 1:** Clinical and radiographic profile of the study participants (n=100)

Findings	Parameter		Present N (%)		Absent N (%)	
Clinical	Pain		98	98%	2	2%
	Swelling	Extraoral	29	29%	71	71%
		Intraoral	82	82%	18	18%
	Abscess		89	89%	11	11%
	Dental Caries		100	100%	0	0%
	Tender on percussion		73	73%	27	27%
Radiographic	Periapical or Furcal involvement		78	78%	22	22%
	Root resorption		73	73%	27	27%

Microorganisms were detected in 68% of the samples, while 32% showed no detectable organisms (p=0.194). Among the samples with microbial presence, Gram-positive organisms accounted for 57% and Gram-negative organisms for 11%, together comprising the total positive isolates. This distribution was statistically significant (p=0.001). Antibiotic susceptibility was observed in 46% of cases, and resistance was present in 29% of samples (Table 2).

**Table 2:** Microorganisms prevalent in the root canals of primary teeth

Variables	Status	No. of samples	Percentage	p-value
Presence of organism	Absent	32	32.00%	0.194
	Present	68	68.00%	
Gram stain	Negative	11	11.00%	0.001
	Positive	57	57.00%	
Presence of susceptibility	Absent	54	54.00%	0.484
	Present	46	46.00%	
Presence of resistance	Absent	71	71.00%	0.001
	Present	29	29.00%	

**Figure 2:**
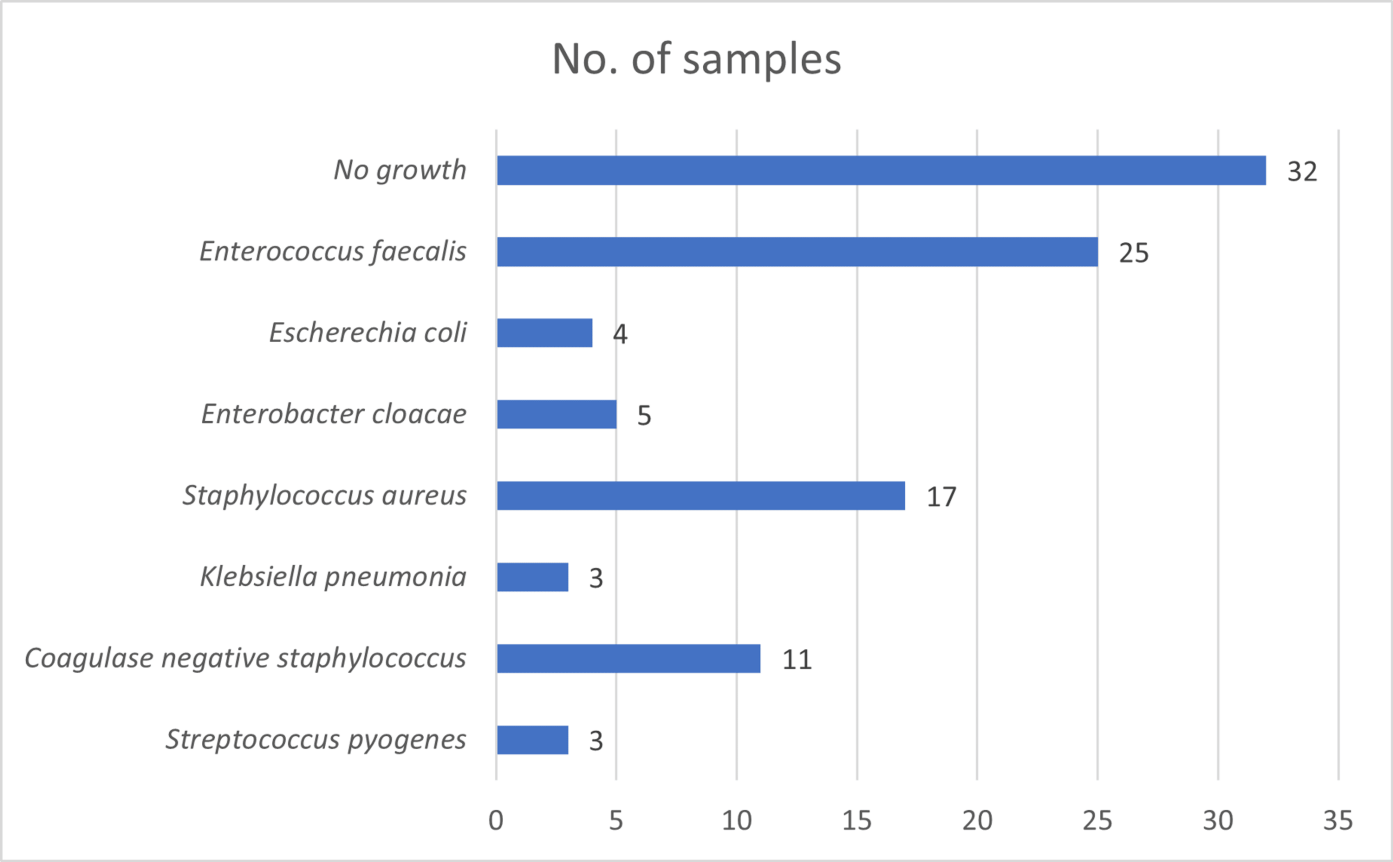
Microbial profile of the sample population

Microbiological analysis showed that Enterococcus faecalis was the most frequently isolated organism (n=25), followed by Staphylococcus aureus (n=17) and coagulase-negative Staphylococcus (n=11). Gram-positive cocci predominated among the isolates, while Gram-negative bacilli were less common, including Enterobacter cloacae, Escherichia coli, and Klebsiella pneumoniae. All the identified organisms were primarily facultative anaerobes, with some exhibiting microaerophilic characteristics. No microbial growth was observed in 32 samples. The overall distribution of microorganisms was statistically significant (p=0.001) (Figure 2).

Antibiotic susceptibility testing revealed that Gram-negative bacilli, E. coli, E. cloacae, and K. pneumoniae, were universally sensitive to amikacin (100%), with high susceptibility also observed for gentamicin and imipenem (75-100%). Piperacillin-tazobactam showed good activity (75-100%), while lower susceptibility was noted for ciprofloxacin, ceftazidime, and amoxicillin-clavulanate, and ceftriaxone demonstrated moderate effectiveness across isolates (Table 3). 

**Table 3:** Antimicrobial susceptibility pattern of Gram-negative bacilli from patients in the study group (n=100)

Antibiotics	Escherichia coli (n=4)	Enterobacter cloacae (n=5)	Klebsiella pneumoniae (n=3)
Amikacin (30 µg)	100%	100%	100%
Gentamicin (10 µg)	100%	100%	75%
Ciprofloxacin (5 µg)	16.7%	40%	70%
Ceftriaxone (30 µg)	55%	60%	65%
Ceftazidime (30 µg)	16.7%	60%	16.7%
Piperacillin Tazobactam (100/10 µg)	100%	80%	75%
Amoxicillin/Clavulanate (20/10 µg)	29%	30%	25%
Imipenem (10 µg)	100%	100%	70%

Among Gram-positive cocci, E. faecalis, S. aureus, and Streptococcus pyogenes were fully sensitive to linezolid and vancomycin (100%), with S. pyogenes also showing complete susceptibility to ampicillin, erythromycin, clindamycin, penicillin, and cotrimoxazole. E. faecalis exhibited moderate susceptibility to ampicillin (33.3%), clindamycin (60%), erythromycin (66.7%), and amoxicillin-clavulanate (54%), while S. aureus showed moderate sensitivity to clindamycin (70.6%), gentamicin (76.5%), and cotrimoxazole (64.7%). Overall, glycopeptides and linezolid were highly effective against Gram-positive isolates, whereas aminoglycosides and piperacillin-tazobactam were most effective for Gram-negative bacteria (Table 4).

**Table 4:** Antimicrobial susceptibility pattern of Gram-positive cocci from patients in study population (n=100) NR: Not recommended (as per Clinical & Laboratory Standards Institute guidelines (CLSI)); NT: Not tested; Vancomycin and Teicoplanin were tested from E strip and Minimum Inhibitory concentration (MIC) were taken as per latest CLSI guidelines; Zero percent carbapenem-resistant Escherichia coli (CRE) and extended-spectrum β-lactamase (ESBL) reported in this study.

Antibiotics	Enterococcus faecalis (n=25)	Staphylococcus aureus (n=17)	Streptococcus pyogenes (n=3)
Ampicillin (10 µg)	33.3%	NR	100%
Clindamycin (2 µg)	60%	70.6%	100%
Erythromycin (15 µg)	66.7%	35.3%	100%
Ciprofloxacin (5 µg)	48%	41.2%	NR
Gentamicin (10 µg)	NR	76.5%	NT
High level Gentamicin (120 µg)	60%	NR	NR
Amoxicillin/Clavulanate (20/10 µg)	54%	52%	59%
Cotrimoxazole (1.25/23.75 µg)	70%	64.7%	100%
Penicillin	NR	NR	100%
Linezolid (30 µg)	100%	100%	100%
Vancomycin (MIC)	100%	100%	100%
Teicoplanin (MIC)	100%	100%	NR

## Discussion

There is a well-established consensus that microorganisms are the main cause of endodontic infections. Of all microorganisms inhabiting the oral cavity, only a few of them can invade the pulp and compromise its function. The presence of pathogenic bacteria is essentially responsible for endodontic infections, leading to pulpitis or pulpal necrosis. These infections comprise mixed microbiota involving commensals, pathogenic aerobes, as well as anaerobic organisms (predominating the pulpal infections) [10]. Studies focusing on profiling microflora involved in endodontic infections provide a key scientific basis to improve clinical practice and endodontic treatment [12]. Antibiotics are frequently prescribed during dental treatments; however, such prescriptions are often not based on accurate clinical indications, such as bacteremia associated with infections. Empirical use without proper identification, cultivation, and in vitro susceptibility testing of the implicated microorganisms has raised significant concerns regarding antimicrobial resistance [13].

Prolonged and unnecessary antibiotic use may also compromise the immune system. Importantly, the oral microbiota serves as a reservoir for multiple antibiotic-resistance genes, including those conferring resistance to commonly used agents such as β-lactams and tetracyclines [14]. Most dental infections can be effectively managed with local interventions such as drainage, extraction, or root canal therapy. Therefore, avoiding unwarranted antibiotic prescriptions is both rational and essential. When systemic therapy is indicated, judicious antibiotic use remains critical for the success of endodontic treatment [13].

The present study evaluated the microbial flora and antimicrobial resistance profiles of microorganisms isolated from primary molars of pediatric patients undergoing pulp therapy. Most endodontic infections are polymicrobial with a predominance of strictly anaerobic bacteria [12]. The microbial spectrum revealed a predominance of Gram-positive cocci, particularly E. faecalis, along with Gram-negative bacilli including E. coli, E. cloacae, and K. pneumoniae. This distribution is consistent with earlier reports demonstrating the persistence of E. faecalis in necrotic pulps and root canals of primary teeth, where it is often associated with treatment resistance and recurrent infections [15].

E. faecalis is a facultative anaerobic bacterium known to cause persistent and recurrent infections. In the present study, it was identified as the most prevalent pathogen. Previous evidence has consistently demonstrated an association between Enterococcus species - particularly E. faecalis - and endodontic infections, underscoring its clinical significance in treatment resistance and failure [16]. The high prevalence of E. faecalis in our study concurs with molecular studies which detected this organism in up to 37.5% of necrotic primary molars using polymerase chain reaction (PCR), compared with 16.7% by culture, highlighting its ability to evade standard microbiological detection [17]. Other investigations have reported its occurrence in 7-12% of root canal samples from deciduous teeth 16, and even in 50% of necrotic pulps when using DNA-DNA hybridization methods [18]. Although traditionally regarded as a pathogen linked with persistent endodontic infections, longitudinal clinical data suggest that its presence alone is not always predictive of pulpectomy failure. In one study with 36-month follow-up, E. faecalis was equally detected in successful and failed cases [19]. Nevertheless, its well-documented virulence traits - including biofilm formation, alkaline tolerance, resistance to intracanal medicaments, and ability to penetrate dentinal tubules -make it a formidable pathogen in pediatric endodontics [20]. Although Gram-negative rods are less common in pediatric endodontic infections than Gram-positive cocci and anaerobes, their isolation in this study is in line with earlier findings. Studies have shown that Klebsiella, Enterobacter, and E. coli can be recovered from infected root canals of deciduous teeth, particularly in advanced or mixed infections [21]. Their presence is clinically relevant given their intrinsic and acquired resistance mechanisms, which may complicate empirical antibiotic selection.

Our findings demonstrated uniform susceptibility of Gram-negative isolates to amikacin and imipenem, while ciprofloxacin and cephalosporins exhibited reduced activity. This mirrors larger surveillance studies which report rising fluoroquinolone and third-generation cephalosporin resistance in Enterobacterales across paediatric populations [22]. Reassuringly, no ESBL-producing or CRE were identified in this cohort, consistent with a community-associated rather than nosocomial resistance ecology [23]. Among Gram-positive isolates, susceptibility to vancomycin, teicoplanin, and linezolid remained 100%, corroborating their preserved efficacy as last-line agents [24]. However, macrolide and clindamycin resistance were frequent, particularly in S. aureus, where erythromycin susceptibility was only 35%. Similar resistance trends have been reported in odontogenic infections, with all E. faecalis isolates resistant to clindamycin, while penicillins retained high activity [25].

Despite these in vitro findings, systemic antibiotics should be prescribed cautiously in pediatric dentistry. Antimicrobial therapy must be rationally guided by the bacterial profile and clinical features of each patient. Clinical reports indicate that antibiotic therapy is often required to manage endodontic infections. β-lactam antibiotics (e.g., penicillin) remain the first-line choice due to their broad effectiveness against endodontic pathogens and favorable safety profile. In cases where penicillin alone fails, β-lactamase inhibitors such as clavulanic acid are combined with amoxicillin to enhance efficacy [14].

The emergence of antibiotic-resistant strains in healthcare is largely attributed to irrational prescribing practices. High resistance levels may be explained by the ability of microorganisms to form biofilms, which hinder antibiotic penetration, and by their capacity to adopt highly protected, phenotypic spore-like states that enhance survival against antibacterial agents [12].

In this study, 68 of the 100 samples demonstrated mixed bacterial growth, while 32 samples showed no growth. The absence of microbial growth, despite clinical and radiographic evidence of infection, may be attributed to the presence of uncultivable microorganisms or strict anaerobes that fail to grow under 5% CO₂ conditions. Although standard sampling protocols were rigorously followed, culture failures remain possible. Importantly, many endodontic infections may be dominated by in vitro non-cultivable bacteria, underscoring the limitations of culture-based methods and highlighting the need for genotypic approaches or advanced microbial profiling techniques to achieve more accurate characterization of the endodontic microbiota [26].

This study provides valuable insight into the antimicrobial resistance patterns of pediatric endodontic isolates in a community setting, with phenotypic confirmation performed in accordance with CLSI guidelines. However, certain limitations must be acknowledged. The relatively small sample size of Gram-negative bacilli restricts the generalizability of findings, and the exclusion of obligate anaerobes-known to play a significant role in endodontic infections-limits the comprehensiveness of the microbial profile.

Future investigations incorporating anaerobic culture techniques and molecular sequencing approaches are warranted to achieve a more complete understanding of the pediatric endodontic microbiota and its resistance determinants. Notably, it is estimated that nearly 50% of microbial species present in infected root canals remain uncultivable, posing significant challenges for identification. Nevertheless, microbiological culture remains essential for evaluating pathogenicity and antimicrobial susceptibility, underscoring the need for complementary genotypic methods to overcome the limitations of conventional culture-based diagnostics.

This study underscores that E. faecalis continues to be a predominant endodontic pathogen in pediatric patients, with occasional isolation of Gram-negative bacilli. First-line access group antibiotics, such as amoxicillin, should remain the preferred systemic therapy when clinically indicated. The absence of ESBL and CRE in this cohort is reassuring; however, sustained surveillance and rigorous antimicrobial stewardship are essential to mitigate the risk of emerging high-level resistance in community-based pediatric dental practice.

## Conclusions

The increasing use and misuse of antibiotics has become a major concern due to the rising prevalence of antimicrobial resistance. In pediatric dental practice, antibiotics are frequently prescribed without microbiological confirmation, which may promote the selection of resistant organisms. Overprescription of these drugs exerts a powerful and consistent selective pressure for resistance, not only in targeted pathogens but also across the patient's entire commensal flora. Dentists must carefully evaluate the characteristics of each infection and prescribe antibiotics judiciously, only when clinically indicated.

A rational approach to antibiotic selection has to be based on scientific data with the continuously evolving flora of orofacial infections. Further genotypic studies are warranted to enhance microbial profiling and resistance detection, thereby strengthening evidence-based practice. Ultimately, awareness of appropriate indications and the rational selection of antibiotics are essential to minimize unnecessary prescriptions and reduce potential adverse effects
